# Anterograde Degeneration along the Visual Pathway after Optic Nerve Injury

**DOI:** 10.1371/journal.pone.0052061

**Published:** 2012-12-26

**Authors:** Yuyi You, Vivek K. Gupta, Stuart L. Graham, Alexander Klistorner

**Affiliations:** 1 Department of Ophthalmology, Australian School of Advanced Medicine, Macquarie University, Sydney, New South Wales, Australia; 2 Save Sight Institute, Sydney University, Sydney, Australia; Dalhousie University, Canada

## Abstract

**Purpose:**

To investigate anterograde degenerative changes along the visual pathway in a rat model of optic nerve axotomy.

**Methods:**

Optic nerve transection was performed in adult Sprague-Dawley rats. Animals were sacrificed at regular time intervals and tissues harvested. Immunoblotting followed by densitometric analysis was used to determine the phosphorylation profile of Akt in the dorsal lateral geniculate nucleus (dLGN) and the primary visual cortex (V1). The neuronal cell size and cell density were measured in the dLGN and the V1 using Nissl staining. The prevalence of apoptosis was characterized by terminal deoxynucleotidyl-transferase-mediated biotin-dUTP nick end labelling (TUNEL) histochemistry. Caspase-3 antibodies were also used to identify apoptotic cells. Neurons and astrocytes were detected using NeuN and glial fibrillary acidic protein (GFAP), respectively.

**Results:**

An early and sustained loss of Akt phosphorylation was observed after optic nerve transection in both dLGN and V1. At week one, a decrease in the neuronal cell size (50.5±4.9 *vs* 60.3±5.0 µm^2^, P = 0.042) and an increase of TUNEL positive cells (7.9±0.6 *vs* 1.4±0.5 ×10^2^ cells/mm^2^, P<0.001) were evident in the dLGN but not in V1. A significant decline in neuronal cell number (14.5±0.1 *vs* 17.4±1.3 ×10^2^ cells/mm^2^, P = 0.048), cell size (42.5±4.3 *vs* 62.1±4.7 µm^2^, P = 0.001) and an increase in apoptotic cells (5.6±0.5 *vs* 2.0±0.4 ×10^2^ cells/mm^2^, P<0.001) appeared in V1 initially at one month post-transection. The changes in the visual pathway continued through two months. Both neuronal cells and GFAP-positive glial cells were affected in this anterograde degeneration along the visual pathway.

**Conclusions:**

Anterograde degeneration along the visual pathway takes place in target relay (LGN) and visual cortex following the optic nerve injury. Apoptosis was observed in both neural and adjacent glial cells. Reduction of Akt phosphorylation preceded cellular and apoptotic changes.

## Introduction

The spread of neurodegeneration [1] is a characteristic feature of diverse neurological disorders, such as Alzheimer’s disease [2], amyotrophic lateral sclerosis [3], Parkinson’s disease [4] and brain trauma [5]. This phenomenon also has been investigated in various animal models, including experimental Alzheimer’s model, brain injuries [5], [6], [7], [8], [9], spinal cord lesions [10], [11] as well as tooth pulp extirpations [12]. The retina and the optic nerve are unique extensions of the central nervous system. In the visual system, both retrograde (visual cortex to retina) [13], [14], [15], [16], [17] and anterograde (retina to visual cortex) [18], [19], [20], [21], [22], [23], [24] spread of degeneration under various pathological conditions has been observed. Insights into anterograde degeneration in glaucoma, which is a leading cause of blindness worldwide, are critical in understanding the pathophysiology of the disease and its impact on the brain [25].

The exact mechanism of the spread of neurodegeneration remains unknown, but programmed cell death has been known to play a major role in it [26]. The involvement of nitrotyrosine induced oxidative injury, glutamate excitotoxicity, cytokine response and more recently synaptic plasticity and redistribution [27], [28] have also been suggested. Recently, tau pathology has been found to spread via the synaptic circuits in transgenic mice with tau expression restricted to a particular region of the brain [29], [30].

Activation of the Akt pathway has been shown to be neuroprotective [31], [32] and has profound effects on synapse number, dendritic plasticity, and circuit function [33]. Cheng et al. [34] have reported the involvement of Akt kinase in suppressing retrograde axonal degeneration. In addition, Kermer et al. [35] revealed the role of insulin-like growth factor (IGF) in protecting retinal ganglion cells (RGCs) via Phosphatidyl inositol 3 kinase (PI3-K) dependent Akt phosphorylation and by inhibition of caspase-3. Akt has a diverse array of cellular protective effects, including cell survival, growth, proliferation, angiogenesis, metabolism, and migration [36]. Akt signalling pathways have also been linked to the production of nitric oxide [37], which can induce oxidative injury as mentioned above. Therefore, the Akt pathway may be involved in the mechanisms of the early signalling change that precede cellular degeneration and apoptosis.

Studying anterograde neurodegeneration in primates [20], [21], [22], [27], [28] is difficult, not only because it takes a relatively long period for neural degeneration to occur in the brain [38], but also because of the inherent anatomy of the primate visual system. In primates, 40% of the axons of RGCs decussate at the chiasm and terminate in layers 1, 4 and 6 of the lateral geniculate nucleus [39]. This poses a real difficulty in isolating the individual layers and their molecular analysis. By contrast, in species with laterally positioned eyes and therefore a relatively small binocular visual field, the proportion of ipsilaterally projecting RGCs tends to be small. Although the rodent visual system shares a lot of similarities with humans, it was estimated that only 2–3% axons of the retinal ganglion cells terminate at the ipsilateral side, whereas over 95% axons project to the contralateral retinorecipient nuclei, which include the superior colliculus (SC) in the midbrain, the suprachiasmatic nucleus (SCN) of the hypothalamus, the dorsal lateral geniculate nucleus (dLGN) of the dorsal thalamus, the ventral lateral geniculate nucleus (vLGN) and the intergeniculate leaflet (IGL) [39], [40]. This provides an ideal model to study anterograde degeneration and to isolate the effects from both eyes. The primary visual cortex (V1) of the rodent (area 17) receives its input predominantly from dLGN, the geniculocortical fibres of which terminate mainly in layer IV of V1 [39].

In the current study, we performed an optic nerve axotomy in rats, and analysed subsequent changes in the Akt activation along with cellular and apoptotic changes in the dLGN and V1 at regular time intervals starting from one week up to two months. The aim of the study was to investigate the mechanisms and extent of anterograde degeneration along the visual pathway.

## Methods

### Animals

All procedures involving animals were conducted in accordance with the Australian Code of Practice for the Care and Use of Animals for Scientific Purposes and the guidelines of the ARVO statement for the Use of Animals in Ophthalmic and Vision Research, and approved by Macquarie University Animal Ethics committee. Male Sprague-Dawley rats with a body weight of 300–350 g (10–12 weeks, Animal Research Centre, Perth) were used. All animals were maintained in an air-conditioned room with controlled temperature (21±2°C) and fixed daily 12-hour light/dark cycles. The animals were anaesthetised with an intraperitoneal injection of ketamine (75 mg/kg) and medetomidine (0.5 mg/kg) for the surgery.

### Optic Nerve Axotomy

The optic nerve was exposed using the surgical protocol described previously in a rat model of optic neuritis [41]. Briefly, the head was shaved and the skin disinfected with 75% ethanol. A 1-cm incision was made in the skin above the orbit of a randomly selected eye. The lacrimal glands and extraocular muscles were resected to expose 3 mm of the optic nerve under an operating microscope. The nerve fibres were completely transected at a distance of 2–3 mm from the globe. In a sham operation, the optic nerve was exposed using the same manipulation, but not cut. Efforts were made to minimise a possible damage to blood vessels during the surgical procedures. The skin incision was sutured and antibiotic administered to prevent infection. Three animals were used for each time point. The animals were allowed to recover from anaesthesia on a warming pad.

### Western Blotting

The dLGN and the V1 (monocular area) were accurately localised and excised from the brain after euthanasia and perfusion with the help of a rat brain slicer under the surgical microscope [42]. The tissue was mixed in lysis buffer (20 mM HEPES, pH 7.4, 1% Triton X-100, 2 mM EDTA) containing protease inhibitors (10 µg/ml aprotinin, 10 µM leupeptin, 1 mM phenymethyl sulfonyl fluoride) and phosphatase inhibitors (1 mM NaVO_3_, 100 mM NaF, 1 mM Na_2_MoO_4_) and sonicated. Insoluble materials were removed by centrifugation at 15,000 g for 10 min at 4°C. Protein concentrations were determined using the BCA protein assay kit (Pierce, Rockford, USA). Samples were then run on a NuPAGE 10% Bis-Tris gel in MOPS SDS running buffer (Invitrogen) and electroblotted to an iBlot gel transfer PVDF membrane (Invitrogen). Next, membranes were blocked in Tris buffered saline (TBS) (20 mM Tris–HCl, pH 7.4, 0.15 M NaCl) containing 5% skimmed milk powder for 1 h. After blocking, membranes were probed overnight at 4°C with primary antibodies (Cell Signalling). Membranes were then washed with TBS buffer and incubated with HRP (horseradish peroxidase)-labeled secondary antibody for 1 h at room temperature. After extensive washing, antibody detection was accomplished with Supersignal West Pico Chemiluminescent substrate (Pierce). Signals were detected using an automated luminescent image analyzer (ImageQuant LAS 4000, GE Healthcare). The densitometric analysis of the band intensities was performed using the ImageJ software (NIH Image) [43].

### Histology

Animals were sacrificed at three different time points post optic nerve axotomy (1 week, 1 month and 2 months; 1 month for the sham group) with an overdose of anaesthetics and then perfused transcardially with 4% paraformaldehyde. For morphological analysis, the brains were fixed in 4% paraformaldehyde overnight, processed in an automatic tissue processor (Leica), and embedded in paraffin. 8-µm thick coronal sections were made using a rotary microtome (Zeiss). Nissl staining and terminal deoxynucleotidyl-transferase-mediated biotin-dUTP nick end labelling (TUNEL) staining (DeadEnd Colorimetric TUNEL System, Promega) were performed on the serial sections to assess cellular morphology and apoptosis, respectively. Cell density was determined for each rat by counting the number of cells in nine standardized microscopic field of 10,000 µm^2^. Cell size was measured by averaging the cross-sectional areas of all the cells in a microscopic field of 40,000 µm^2^ (AxioVision, Carl Zeiss). All histological analysis was performed on the ventrolateral part of the dLGN and the layer IV of monocular V1. For immunohistochemistry study, cryosections (15 µm) were incubated with NeuN/Caspase-3 or GFAP/Caspase-3 antibodies (1∶100), diluted in 4% normal horse serum overnight at 4°C, and subsequently subjected to incubation with Alexa-Fluor 488 and Alexa-Fluor 594 (1∶100 in TPBS) for 1 hour in dark. Immunostained sections were then analysed using confocal microscope (DM-6000, Leica).

### Data Analysis

The Akt phosphorylation, cell density, cell size and number of apoptotic cells from the contralateral dLGN and V1 were compared with those from the ipsilateral side of the sectioned optic nerve using the Student’s t test. The data were plotted using Graphpad Prism software (5.0). A *p* value of <0.05 was considered statistically significant.

## Results

### Akt Phosphorylation

**Figure 1:**
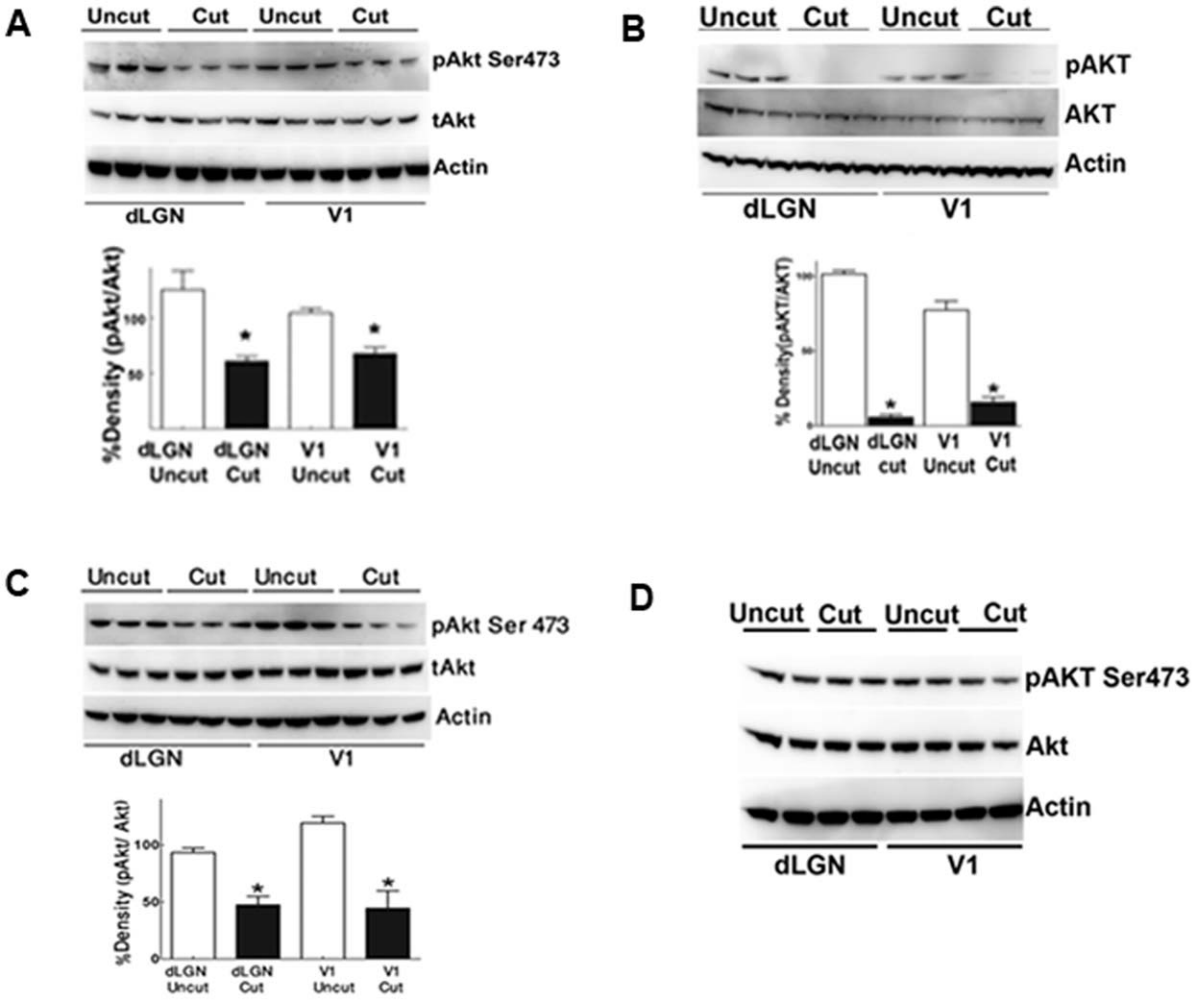
Western blots show the expression of pAkt/Akt in the dLGN and V1 from week 1 to month 2 (n = 3 for each time point, n = 2 for the sham). Actin was used as the loading control. Error bars represent SEM. (A) 1 week; (B) 1 month; (C) 2 months; (D) Sham.

The phosphorylation of Akt at Ser473 residue has been shown to be important for its activation. Western blotting using Akt specific phospho antibodies demonstrated a decline of the Akt phosphorylation level in the dLGN as well as the visual cortex starting from the week 1 time point after optic nerve axotomy (Figure 1A). The densitometric ratios of phosphoAkt/total Akt in the dLGN and V1 are shown. Actin was used as the loading control in each case. The decrease of phosphorylation was already evident in both dLGN and V1 at week 1 with more significant decline by the end of first month (Figure 1B). This loss was partially compensated at 2 months (Figure 1C), with pAkt levels gradually rising and being comparable to that observed at week 1 post optic nerve axotomy.

### Cell Density and Cell Size

**Figure 2:**
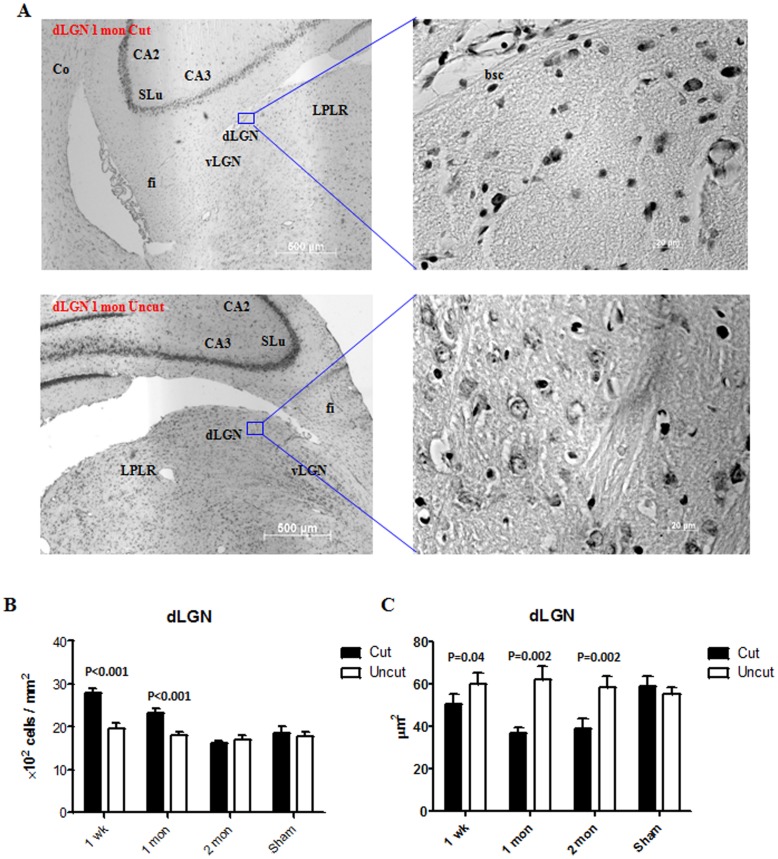
Histological changes in the dLGN. (A) Representative sections (Nissl staining) of the dLGN at month 1 after optic nerve transection. (B) Changes in the cell density in the dLGN from week 1 to month 2. (C) Changes in the cell size (cross-sectional area) in the dLGN from week 1 to month 2. (n = 3 for each time point; n = 2 for the sham control). Error bars represent SEM. dLGN: dorsal lateral geniculate nucleus; vLGN: ventral lateral geniculate nucleus; LPLR: lateral posterior thalamus nucleus, laterorostral part; fi: fimbria of the hippocampus; CA2: field CA2 of the hippocampus; CA3: field CA2 of the hippocampus; Co: cortex.

**Figure 3:**
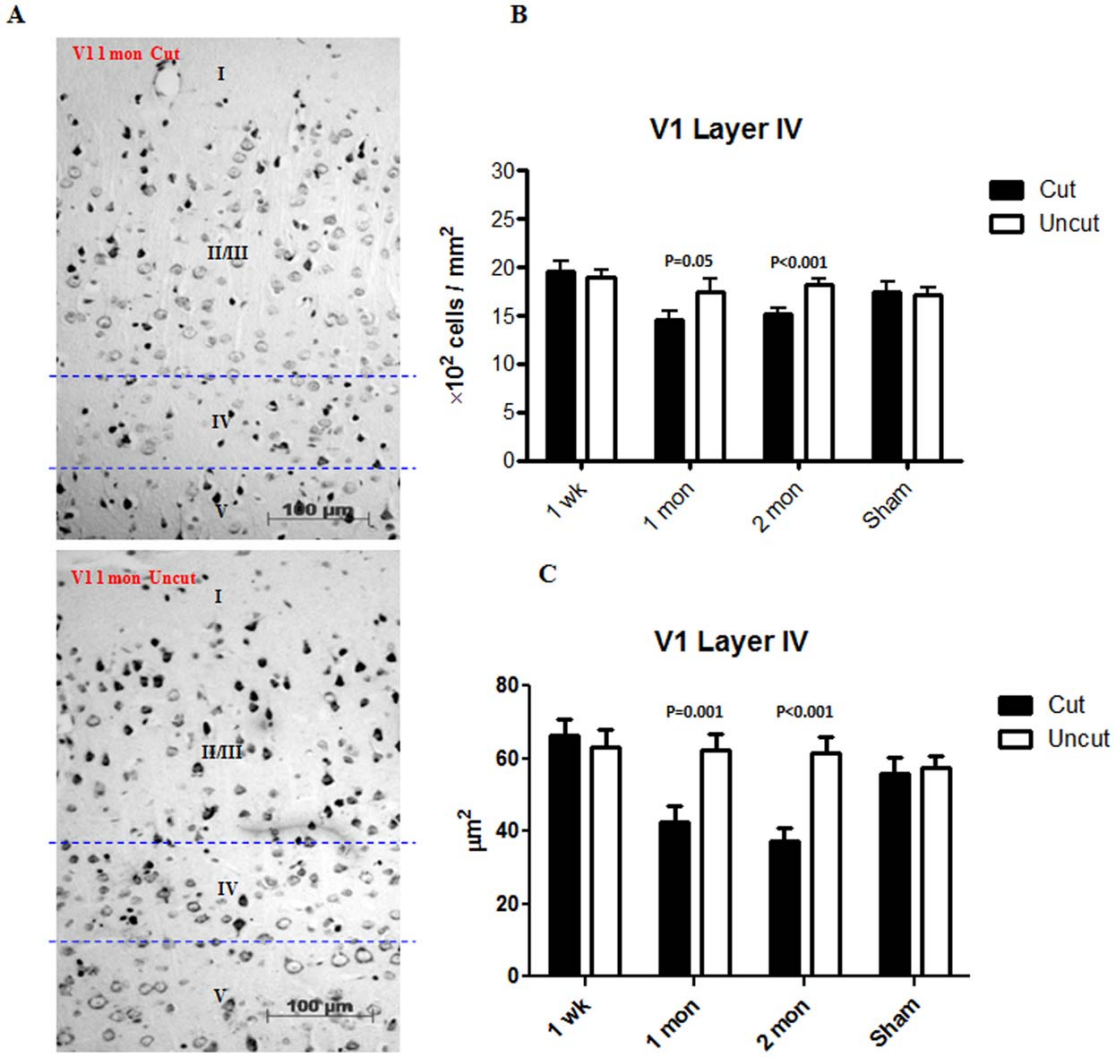
Histological changes in V1. (A) Representative sections (Nissl staining) of V1 from a rat sacrificed at month 1. (B) Changes in the cell density in layer IV of V1 from week 1 to month 2. (C) Changes in the cell size in layer IV of V1 from week 1 to month 2. (n = 3 for each time point; n = 2 for the sham control). Error bars represent SEM.

The changes in the cell density and size are important indicators of the morphological changes caused by tissue stress. Figure 2A shows representative images of the rat dLGN 1 month after the optic nerve axotomy. The ipslilateral side was used for comparison in each case. The measurements of cell density and cell size in the dLGN from week 1 to month 2 are shown (Figure 2B, C). An initial increase in cell density was observed in the dLGN at week 1 and month 1 following axotomy, which subsided to normal level and became comparable to the control side at month 2. There was a significant reduction of cell size in the dLGN at week 1 which progressively decreased through 1 month time point. Representative images of cell size and cell density measurements of V1 are presented in Figure 3A with the ipsilateral side as control. No change in cell density or size was observed at week 1 in layer IV of V1 (Figure 3B, C). Cell density as well as cell size, however, started to decrease from 1 month and this tendency persisted through the 2 months time period.

### Apoptosis

**Figure 4:**
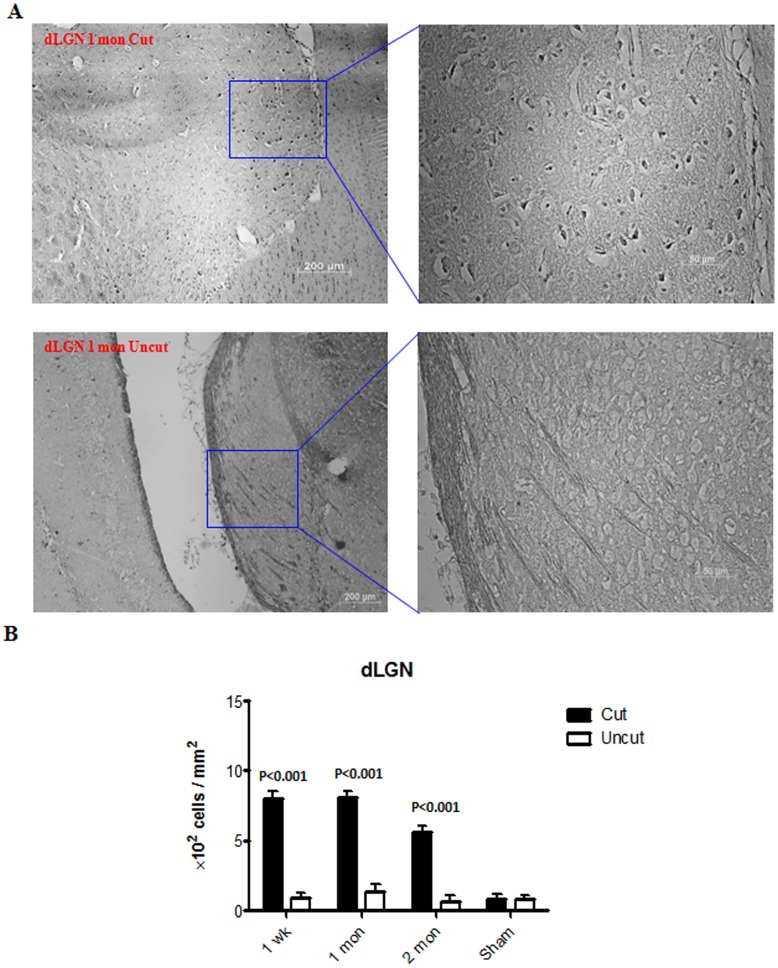
Apoptotic changes in dLGN. (A) Representative TUNEL staining of the dLGN on month 1. (B) A plot of density of TUNEL positive cells in the dLGN from week 1 to month 2. (n = 3 for each time point; n = 2 for the sham control). Error bars represent SEM.

**Figure 5:**
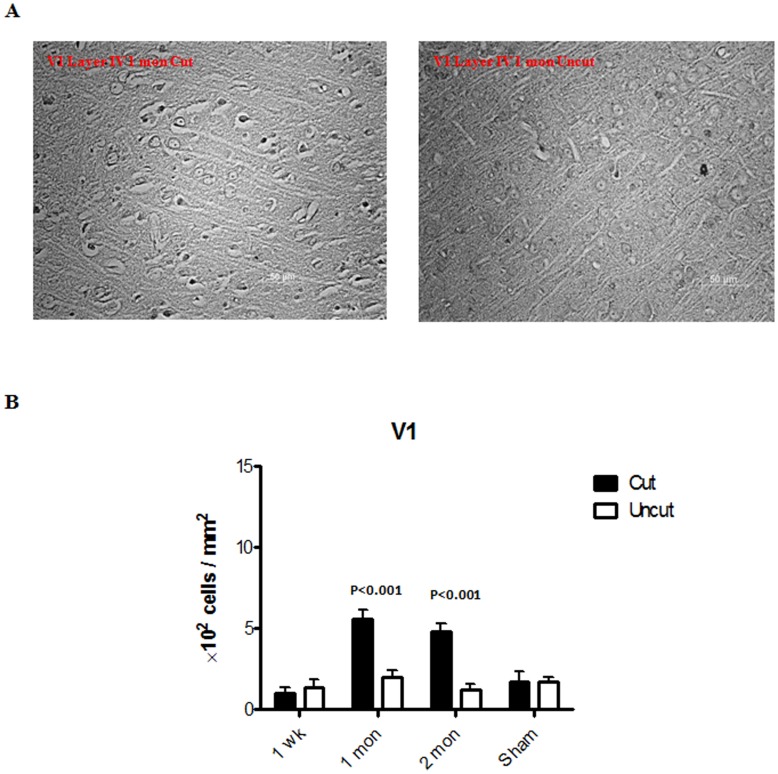
Apoptotic changes in V1. (A) Representative TUNEL staining of layer IV of V1 on month 1. (B) Density of TUNEL positive cells in layer IV of V1 from week 1 to month 2. (n = 3 for each time point; n = 2 for the sham control). Error bars represent SEM.

**Figure 6:**
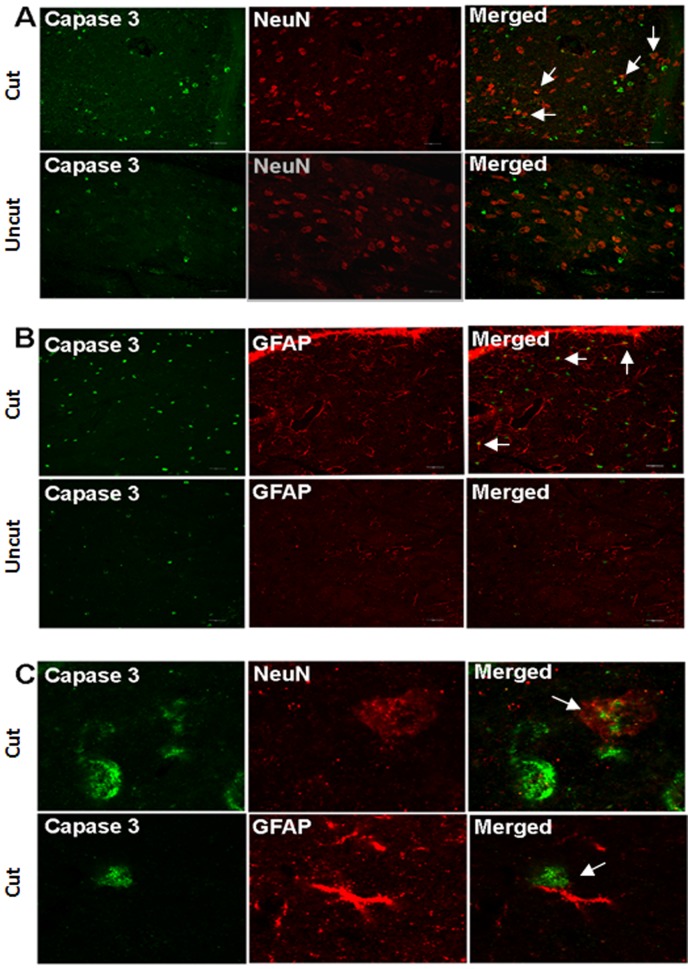
Immunostaining of the dLGN using Caspase 3/NeuN and Caspase 3/GFAP antibodies. Both NeuN –positive neurons and GFAP-positive glial cells undergo apoptosis in the dLGN. (A) Double staining of Caspase 3 and NeuN. Arrows indicate apoptotic neurons. (B) Double staining of Caspase 3 and GFAP. Arrows indicate apoptotic glial cells. (C) High magnification of the confocal microscope shows co-localisation of Caspase 3/NeuN and Caspase 3/GFAP in individual cells.

**Figure 7:**
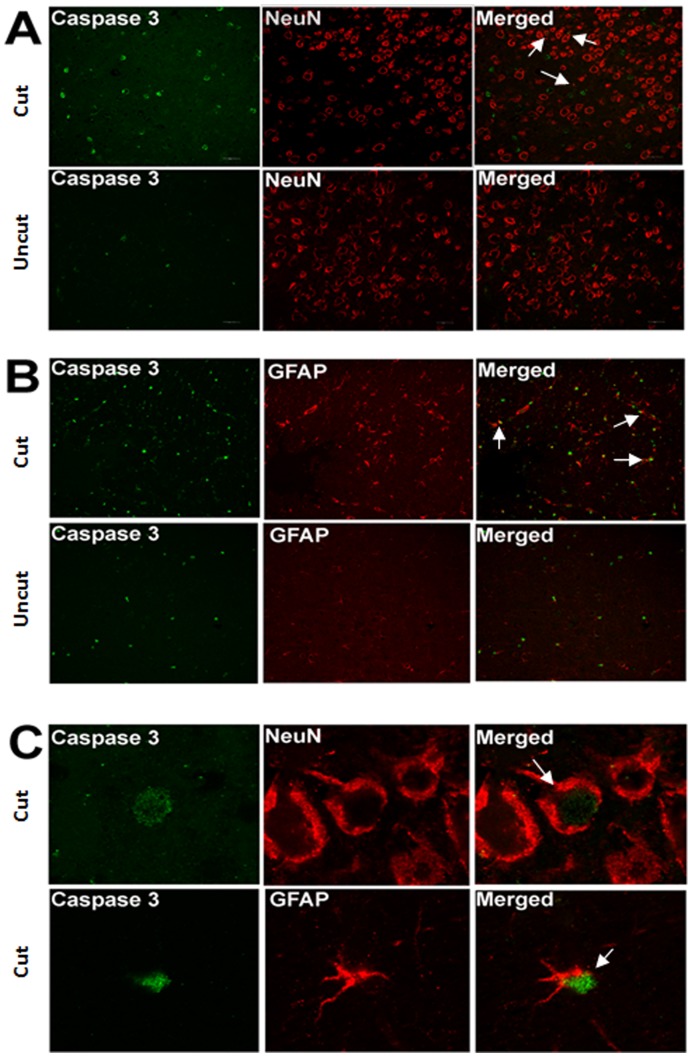
Immunostaining of layer IV of V1 using Caspase 3/NeuN and Caspase 3/GFAP antibodies. (A) Double staining of Caspase 3 and NeuN. Arrows indicate apoptotic neurons. (B) Double staining of Caspase 3 and GFAP. Arrows indicate apoptotic glial cells. (C) Co-localisation of Caspase 3/NeuN and Caspase 3/GFAP in individual cells.

A significant increase in the number of TUNEL positive cells was observed in the dLGN sections as early as week 1 (Figure 4B). This remained significant at later time points (the apoptotic staining in the dLGN, however, was observed to decrease at month 2). Parallel to the observations in cellular size and density, no apoptotic changes were observed at week 1 in V1 following the optic nerve transection (Figure 5B). The representative images of TUNEL staining for the dLGN and V1 at one month are presented (Figure 4A, 5A). A significant increase in the number of TUNEL positive cells was observed in V1 at 1 month, which also remained significant at the 2 month time point. However, the extent of apoptotic staining observed in V1 was lower compared to that of the dLGN. In accordance with TUNEL staining results, the dLGN and V1 on the contralateral side of the axotomized optic nerve depicted an increased expression of the cleaved caspase-3 compared to the ipsilateral side (Figure 6, 7). Immunohistochemistry of NeuN/Caspase-3 and GFAP/Caspase-3 further demonstrated that both NeuN-positive neuronal cells (Figure 6A, 7A) and GFAP-positive glial cells (Figure 6B, 7B) in the dLGN and V1 were affected by apoptosis. High resolution confocal imaging revealed that caspase-3 expression colocalised with NeuN in neuronal cells as well with GFAP in GFAP expressing glial cell populations (Figure 6C, 7C). The caspase staining levels were 3–4 fold higher in the dLGN at one month post optic nerve section whereas in V1 the apoptotic staining was 2–2.5 fold more compared to that of the ipsilateral side.

## Discussion

Several recent studies highlight the importance of the spread of neuronal cell degeneration to distant regions in neurodegenerative diseases [1], [2], [3], [5], [6], [7], [8], [9], [10], [11], [12]. Yücel and co-workers described anterograde degeneration along the visual pathway in primate glaucoma models as well as glaucoma patients [22], [24]. However, little is known about the molecular and cellular changes involved in the spread of neuronal degeneration to LGN and visual cortex upon optic nerve damage. In this study, we investigated secondary degenerative changes in the brain using a rat model of optic nerve axotomy. Secondary degeneration was first observed in the dLGN as early as 1 week after the optic nerve transection, which was consistent with the previous studies [44], [45]. The axons of the second order dLGN neurons synaptically connect with the third order neurons in the visual cortex. Our study demonstrated significant histological changes in layer IV of V1 in rats upon acute optic nerve damage. This implies a degenerative effect spreading to at least a third order of neuron along the visual pathway via two synaptic connections.

A significant decline in the Akt activation in both dLGN and V1 was observed one week after optic nerve transection. This loss was maintained during the entire follow up period. The decrease of Akt phosphorylation level was observed prior to any detectable histological and apoptotic changes in V1, which suggested that dephosphorylation of Akt is an early indicator of degenerative changes in the cells [31], [32], [34], [35], [36], [46], [47]. Changes in the phosphorylation pattern of Akt can be an important indicator of the initial molecular changes preceding degenerative effects in the spread of neuronal degeneration. Akt, which is located at the cross-roads of insulin receptor (IR), insulin-like growth factor 1 receptor (IGF-1R) and other important signalling pathways, can mark the cumulative effect of signalling cascades during the initial stages of cell apoptosis. Activation of Akt also plays a vital role in neuronal survival and maintenance of normal cellular structure and function.

It is not known how the loss of Akt signalling eventually leads to apoptotic changes in anterograde degeneration. We did not observe any differences in the phosphorylation status of downstream effector Glycogen synthase kinase 3 beta (GSK3β) (Figure S1), indicating that the effects of the loss of Akt signalling are transmitted to the cellular apoptotic machinery through an alternative signalling pathway. It is important to mention that the loss of Akt phosphorylation at 1 month after optic nerve transection was more evident compared to that observed at 1 week, and this loss was then partially restored at two months time point. Since Akt is a critical downstream target of several important signalling pathways, it is likely that its loss is compensated through activation/up-regulation of some other upstream molecules.

A paradoxical increase in the cell density was observed in the dLGN 1 week post optic nerve transection. A plausible explanation for this increase is that the dLGN tissue itself undergoes a certain degree of atrophy, which arbitrarily increases the cell density [44]. As expected, we also observed a reduction in the size of the dLGN on the contralateral side of the axotomized optic nerve compared to that on the ipsilateral side. The apparent increase in cell number was gradually resolved at 2 months time-point post transection. This can potentially be attributed to the onset of significant amount of apoptosis and resulting cell loss. However, no such increase in cell density was observed in layer IV of V1, indicating that effects of tissue atrophy in V1 were not as profound and obvious as in the dLGN. The tissue specific atrophy in the dLGN may be attributed firstly, to the relatively higher proportion of apoptotic cells in the dLGN (Figure 4, 5), as V1 is spatially distant to the injury site and located secondary to the dLGN. Secondly, the V1 tissue is anatomically larger in size and layer IV is embedded in and supported by other cortical layers, making it difficult to observe any tissue shrinkage effect. Neuronal shrinkage is a well-known cellular response to pathological injury, and a reduction of soma size corresponds to a decline of neuronal function [22]. It was observed that the neurons in the contralateral dLGN and V1 were smaller than those in the ipsilateral side of the cut optic nerve. This decrease in cell size was first evident in the dLGN at week 1 and then became significant in V1 at one month, indicating that anterograde degeneration gradually progressed and was transmitted along the visual pathway from the relay centre to the cortical centre.

The loss of cell size and cell number was translated into an increased apoptosis in the dLGN and V1 tissues corresponding to the axotomized optic nerve. Interestingly, TUNEL positive cells were observed in both dLGN and V1, contrary to the observations of Zhang et al. [44] who used a chronic model of ocular hypertension. It is possible that the anterograde apoptotic changes are quite subtle and not detectable in a chronic model of glaucomatous damage. Similar to the progression of changes in the cell size and cell number along the visual pathway, no significant apoptosis was observed in V1 until one month post optic nerve transection. These observations indicate that secondary degeneration which is accompanied by cell death progresses gradually along the visual pathway. These results further raise a possibility that certain mild intricate changes may take place in the secondary visual cortex and other structurally and functionally inter-linked regions, but as yet remain undetected due to their subtle nature, limitations of the experimental techniques and associated complexity of inter-regional connections within the brain. Studies are in progress in our laboratory to identify the extent of the spread of degeneration in other parts of the brain.

Immunohistochemical observations further indicated that both neuronal and glial cells are affected in anterograde degeneration along the visual pathway. Zhang et al. [44] observed similar results and showed the involvement of glial cell activation in secondary neuronal degeneration in the SC and dLGN. While direct evidence of synaptic involvement in glial cell degeneration is lacking, there is a possibility that nitric oxide released from apoptotic retinal ganglion cells may diffuse along the axons in the form of peroxynitirte (ONOO^−^) radicals, and induce secondary neuronal as well as glial cell death [48]. It would be helpful to use trans-synaptic tracers [49] to identify the synaptic connectivity between the primary apoptotic neurons and the subsequently affected neurons.

In conclusion, this study established the occurrence and extent of anterograde degeneration in the dLGN and V1 along the visual pathway upon optic nerve injury, and revealed substantial Akt deactivation. This study also highlights the importance of sufficient Akt phosphorylation maintenance in higher visual centers of the brain as a potential way of neuroprotection.

## Supporting Information

Figure S1 **Western blots show the expression of pGSK3β/GSK3β in the dorsal lateral geniculate nucleus (dLGN) and the primary visual cortex (V1) on week 1 and month 2 (n = 3 for each time point).**
(TIF)
