# Supplementary figures and images for: Anterograde Degeneration along the Visual Pathway after Optic Nerve Injury
Source: PLoS One. 2012 Dec 26;7(12):e52061. doi: 10.1371/journal.pone.0052061 (PMC3530579; doi:10.1371/journal.pone.0052061)

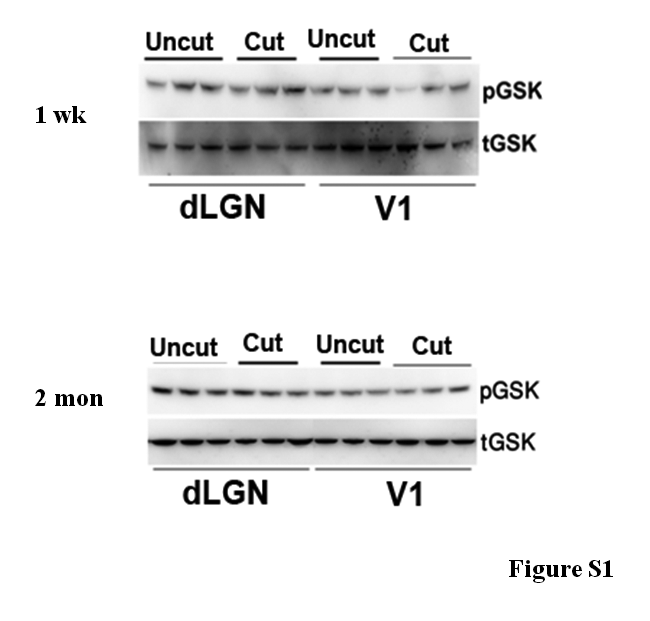

Supplement: Figure S1 — Western blots show the expression of pGSK3β/GSK3β in the dorsal lateral geniculate nucleus (dLGN) and the primary visual cortex (V1) on week 1 and month 2 (n = 3 for each time point). (TIF) [file pone.0052061.s001.tif]
